# Structure and collagen crimp patterns of functionally distinct equine tendons, revealed by quantitative polarised light microscopy (qPLM)

**DOI:** 10.1016/j.actbio.2018.01.034

**Published:** 2018-04-01

**Authors:** Ewa M. Spiesz, Chavaunne T. Thorpe, Philipp J. Thurner, Hazel R.C. Screen

**Affiliations:** a School of Engineering and Materials Science, Queen Mary University of London, Mile End Rd, London E1 4NS, United Kingdom; b Department of Bionanoscience, Kavli Institute of Nanoscience, Delft University of Technology, Van der Maasweg 9, 2629 HZ Delft, The Netherlands; c Comparative Biomedical Sciences, Royal Veterinary College, Royal College Street, London NW1 0TU, United Kingdom; d Institute of Lightweight Design and Structural Biomechanics, TU Wien, Getreidemarkt 9, A-1060 Vienna, Austria

**Keywords:** Tendon, Collagen crimp, Quantitative polarised light microscopy (qPLM), Birefringence, Fascicles, Interfascicular matrix (endotenon)

## Abstract

Structure-function relationships in tendons are directly influenced by the arrangement of collagen fibres. However, the details of such arrangements in functionally distinct tendons remain obscure. This study demonstrates the use of quantitative polarised light microscopy (qPLM) to identify structural differences in two major tendon compartments at the mesoscale: fascicles and interfascicular matrix (IFM). It contrasts functionally distinct positional and energy storing tendons, and considers changes with age. Of particular note, the technique facilitates the analysis of crimp parameters, in which cutting direction artefact can be accounted for and eliminated, enabling the first detailed analysis of crimp parameters across functionally distinct tendons.

IFM shows lower birefringence (0.0013 ± 0.0001 [−]), as compared to fascicles (0.0044 ± 0.0005 [−]), indicating that the volume fraction of fibres must be substantially lower in the IFM. Interestingly, no evidence of distinct fibre directional dispersions between equine energy storing superficial digital flexor tendons (SDFTs) and positional common digital extensor tendons (CDETs) were noted, suggesting either more subtle structural differences between tendon types or changes focused in the non-collagenous components.

By contrast, collagen crimp characteristics are strongly tendon type specific, indicating crimp specialisation is crucial in the respective mechanical function. SDFTs showed much finer crimp (21.1 ± 5.5 µm) than positional CDETs (135.4 ± 20.1 µm). Further, tendon crimp was finer in injured tendon, as compared to its healthy equivalents. Crimp angle differed strongly between tendon types as well, with average of 6.5 ± 1.4° in SDFTs and 13.1 ± 2.0° in CDETs, highlighting a substantially tighter crimp in the SDFT, likely contributing to its effective recoil capacity.

**Statement of Significance:**

This is the first study to quantify birefringence in fascicles and interfascicular matrix of functionally distinct energy storing and positional tendons. It adopts a novel method – quantitative polarised light microscopy (qPLM) to measure collagen crimp angle, avoiding artefacts related to the direction of histological sectioning, and provides the first direct comparison of crimp characteristics of functionally distinct tendons of various ages.

A comparison of matched picrosirius red stained and unstained tendons sections identified non-homogenous staining effects, and leads us to recommend that only unstained sections are analysed in the quantitative manner.

qPLM is successfully used to assess birefringence in soft tissue sections, offering a promising tool for investigating the structural arrangements of fibres in (soft) tissues and other composite materials.

## Graphical abstract

**Figure:**
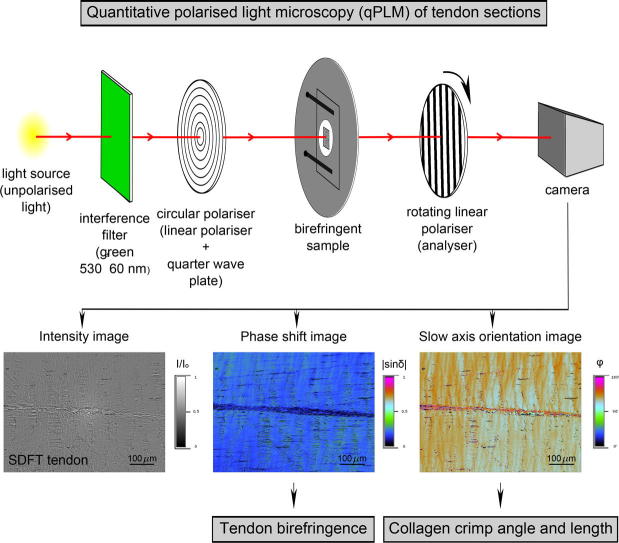

## 1 Introduction

The structure of complex materials is closely related to their function and mechanical properties and vice versa. Collagenous tissues are no exception, therefore there is considerable interest in understanding the mechanical performance of tissues, based on studying the details of their composition and structural arrangement. Tendon is a highly ordered unidirectional fibrous composite, consisting mainly of hierarchically organised collagen type I molecules. Collagen is the most abundant protein and main load-bearing constituent of tendons and many other tissues. It attains its mechanical properties through hierarchical assembly. In tendon this spans from the tropocollagen triple helix at the molecular level, to collagen fibrils, fibres, fascicles, fascicle bundles and whole tendons [1]. At each hierarchical level the collagenous matrix is interspersed with a non-collagenous matrix, rich in proteoglycans [2].

**Fig. 1:**
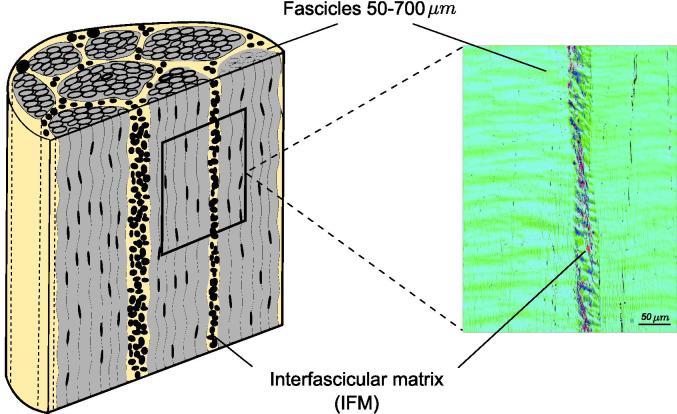
Schematic of an equine superficial digital flexor tendon (SDFT) with an unstained histological section showing the fascicles and the interfascicular matrix (IFM) imaged using quantitative polarised light microscopy (qPLM). The two phases of the tendon mesostructure are clearly differentiated.

Because of its simple structure relative to other connective tissues, tendon tissue is often treated as a calibration material for a variety of techniques [3], [4]. However, despite this appearance of relative structural simplicity [5], mesoscopically the tissue is inhomogeneous, featuring two different regions: fascicles, occupying most of the tendon volume, and interfascicular matrix (IFM; also referred to as the endotenon), between and interconnecting the fascicles, see Fig. 1. Fascicles are mainly composed of uniaxially arranged collagen fibrils, predominantly consisting of collagen type I, with smaller amounts of collagen type III, while the interfascicular matrix is a less dense and less organized region, containing less collagen type I, and greater amounts of collagen type III, proteoglycans and elastin [6], [7].

A series of recent studies have focused on understanding the composition and function of fascicles and their connecting interfascicular matrix [6], [7], [8], [9]. These studies have shown that the structure and composition of tendons differs in order to meet their different functional needs, highlighting functional distinctions between energy storing and positional tendons. Energy storing tendons, such as the human Achilles or equine superficial digital flexor tendon (SDFT), are specialised to withstand the high strains they encounter in-vivo (over 10% and 16%, respectively [10]). By contrast, positional tendons, like the human anterior tibialis or equine common digital extensor tendon (CDET), are specialised for placement of a limb and encounter much lower strains in-vivo (approximately 3% [10]). Recent data have indicated that the IFM in particular differs in these two tendon types. The IFM in energy storing tendons is more fatigue resistant than the IFM in positional tendons, highlighting tendon specialisation to meet functional needs [10], [11], [12]. However, complete understanding of the structural organisation of collagen fibres within fascicles and the IFM in different tendon types remains obscure.

In addition to the composition and structural arrangement of fibres, other features, like collagen fibre crimp morphology, may differ between functionally distinct tendons, and therefore may have a crucial impact on tendon mechanical behaviour [12], [13], [14]. Commonly, estimates of crimp angle and length are measured from 2D histological sections of tendons. While measurements of crimp length are independent of tendon sectioning plane, measurements of crimp angle depend strongly on the relationship between the cutting plane and the crimp propagation plane [15], and therefore the analysis of crimp angle from 2D histological sections may result in large errors. More detailed quantitative characterisation of 3D crimp angle would be of significant benefit for the mechanical modelling of collagenous structures within tendons, as well as for general understanding of structure-function relationships in tendon.

Multiple observations of biological samples with polarized light have been used [16], [17], often in a non-quantitative manner [18], [19], [20], [21], for revealing the structure and organization of tissue components. Briefly, this technique involves placing a thin section of a sample between a polarizer and an analyser, and observing how the constituents of the sample change the polarization state of the transmitted light. In order to change the polarization state of the light, the constituents of the sample must be birefringent, or optically anisotropic. This will cause a split of the incident polarized light into two rays, travelling through the material with different velocities due to different refractive indices in these directions: the ordinary ray (or fast ray, in the direction with a lower refractive index) and the extraordinary ray (or slow ray, in the direction with a higher refractive index). Recombination of the two rays upon exiting the material results in a phase shift or retardation of one ray with respect to the other [22].

There are two types of birefringence that contribute to the total birefringence observed in a sample: intrinsic and form birefringence. Birefringence resulting from the molecular bonds in a material is called the intrinsic birefringence. Collagen, the major component of most tissues, is highly intrinsically birefringent, which allows for direct observation of collagenous tissues with polarized light. The amount of intrinsic collagen birefringence can be related to the amount (or volume fraction) of collagen fibres in the imaged section. Form birefringence relates to the spatial arrangement of a fibrillar structure, and can also be observed in collagenous tissues under polarized light. Form birefringence of collagenous structures is related to the spatial dispersion of collagen fibres (tissue anisotropy). The tissue total birefringence measured with polarized light is a combination of the intrinsic birefringence of its birefringent components and their arrangement in space. Adopting polarised light analysis tools, fibrillar arrangement has successfully been evaluated in hard [3], [23], [24] and soft tissues [25], [26], [27], [28]. In principle, tissues do not require staining in order to visualise the collagen fibre arrangement using polarised light, however contrast agents have been frequently used to augment the birefringence, particularly in soft tissues. The most commonly used stain is picrosirius red, which is an acidic stain that contains six sulfonate groups interacting with collagen molecules that are rich in basic amino acids [25], [26], [29], [30], [31].

A common problem when analysing collagen arrangement with polarised light is the issue of fibres altering their appearance when observed with the polariser at different angles [32], [33], [34]. To help overcome this, and facilitate a quantitative assessment of birefringent samples, the quantitative polarised light (qPLM) technique has been developed [3], [35], [36], [37]. The qPLM technique offers a quantitative assessment of samples’ total birefringence and therefore information about 3D (out-of-plane) structural ordering, that is reflected in the optical anisotropy. Additionally, qPLM reports the orientation of the slow axis of a material (the direction with the highest refractive index), from which morphological parameters can be evaluated in the observed tissue sections, such as the in-plane arrangement of collagenous fibres and crimp characteristics. The combination of the out-of-plane and in-plane arrangements, both related to total birefringence, allow not only for quantitative assessment of birefringence, but can also be used to alleviate the effects of sectioning plane on measured structural characteristics, like crimp angle. To avoid underestimation, crimp angle should be analysed only in samples cut directly parallel with the crimp propagation axis, hence qPLM can be used to determine the plane of a sample relative to that of crimp and exclude any samples which are not sectioned parallel with the crimp propagation axis. This allows one to explore the characteristics of collagen crimp, and contributes new insight into crimp arrangement, helping address the debate as to whether crimp is a planar wave [38], with a variation of angles and lengths within and between fascicles [39] or a 3D (helical) wave [40].

In this study, we used quantitative polarised light microscopy to investigate morphological features of collagen fibres in functionally distinct equine tendons: the energy storing superficial digital flexor tendon (SDFT) and positional common digital extensor tendon (CDET) in order to identify potential differences in fibre arrangement and collagen crimp across fascicles and the IFM of these functionally distinct tendons, and the optical anisotropy in fascicles and interfascicular matrix, reflecting the distinct composition and collagen fibre arrangements within these regions. We investigated if aging would result in altered fibre arrangements and altered collagen crimp patterns in both tendon types. Finally, we tested if birefringence patterns and relative signal magnitude are preserved with staining in site-matched picrosirius red stained and unstained tendon sections.

## 2 Materials and methods

### 2.1 Tissues, sample preparation and staining

The superficial digital flexor tendon (SDFT) and common digital extensor tendon (CDET) were dissected from a single forelimb of 6 horses directly post mortem, all acquired as waste material from a licensed abattoir. None of these twelve tendons exhibited macroscopic evidence of injury. Horses were divided into two age groups: three young horses aged 3–7 years and three old horses aged 17–20 years. Additionally, a visibly injured SDFT of a 17 years old horse was also dissected and considered independently. A sample (approximately 10 mm × 5 mm × 5 mm) was cut from the core of the mid-metacarpal region of each of the 13 tendons, ensuring a matching region of each tendon was assessed and that this encompassed the region of damage in the injured tendon.

Samples were prepared following standard histological preparation methods, first fixed in 4% paraformaldehyde in phosphate-buffered saline (PBS) for 48 h at 4 °C, then stored in 70% ethanol for 24 h, followed by processing to paraffin wax blocks. Six 5 µm-thin longitudinal sections were cut from each paraffin block and mounted on glass slides, after which three sections from each block were stained with picrosirius red using standard protocols [25], [26], [41], while the other three sections were left unstained (total of 78 sections for analysis). Fixed, paraffin embedded sections were adopted over frozen sections, to ensure a constant thickness within a section, necessary for the quantitative evaluation of birefringence using the qPLM method. Standard 5 µm thick histology sections were adopted as these resulted in sufficient signal to be processed utilising the qPLM method. Thicker sections could be adopted, keeping in mind that these may result in obtaining higher order phase shift signals and/or result in image blurring while imaging numerous collagen fibres within the light path.

### 2.2 Quantitative polarised light microscopy (qPLM)

**Fig. 2:**
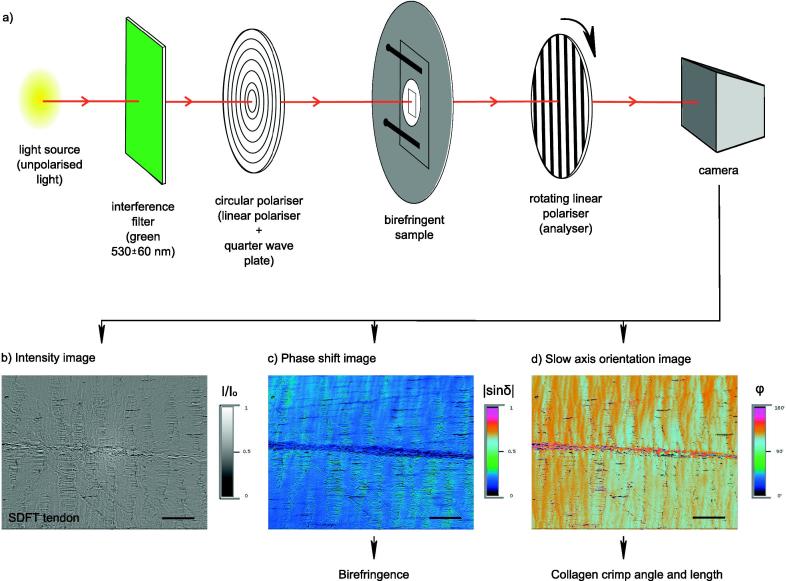
(a) Schematic of the quantitative polarised light microscopy (qPLM) system used in the study. The qPLM technique outputs three matching images: a grey scale intensity image I/I_0_ that can be used to distinguish tendon zones (fascicles and the interfascicular matrix) (b), a false-coloured image of the phase shift |sin δ| that is directly related to tendon birefringence which in turn can be related to the directional dispersion of collagen fibres in a tissue (c) and a false-coloured slow axis orientation image *φ* (d) intensifying the visibility of collagen crimp and facilitating fascicle crimp length measurements. Moreover the combination of c) and d) enables collagen crimp angle measurements without the sectioning artefact resulting from the unknown relationship between the sectioning and crimp propagation planes (details in 2.3, 2.4). The typical images from the three channels shown above were taken from an unstained SDFT section of a young horse (b–d). Scale bars = 100 µm.

Samples were imaged using a circularly polarised light technique, also referred to as quantitative polarised light microscopy (qPLM) [3], [23], [27], [28], [42]. The experimental set-up for the measurements utilised a regular polarised light microscope (Axioscope 40, Carl Zeiss, Goettingen Germany), an interference filter (green, 530 ± 60 nm), a circular polariser (consisting of a linear polariser and a quarter wave plate), a rotating linear polariser (analyser) and a camera (AmScope MT**,** Irvine, CA, USA). Both polarisers were manufactured by Werner Kaminsky, University of Washington. It should be noted that a conventional microscope can be easily converted into a (quantitative) polarised light microscope by placing these specific filters in the light path (as long as the software to manage overlapping images collected at various angles of the rotating polariser is additionally available). A schematic showing the arrangement of the components and details of the subsequent light path through the system, alongside typical output images collected from an unstained 5 µm-thick tendon section are shown in Fig. 2. All images were captured at 20× magnification using the Rotopol software (Werner Kaminsky, University of Washington), and the scale bar was calibrated with a stage micrometer, resulting in a pixel size of 0.23 µm. As qPLM is a transmitted light technique, its resolution in the z direction is limited to the thickness of sample investigated (here 5 µm). The resolution (diffraction limit) in the x-y directions was approximately 1 µm at the magnification used.

The system minimises imaging and polarisation artefacts in birefringence measurements [3], [24], [36], [43], because the information is obtained from three independent imaging channels: light intensity (I/I_0_, see Fig. 2b), the phase shift |sin δ| (Fig. 2c) and orientation angle of the slow axis of material *φ* (Fig. 2d). The outputs of the phase shift and slow axis orientation channels and their relationship to tendon morphometric parameters are described in detail in the two following sections (2.3, 2.4). A region of interest was randomly selected in each tendon section, ensuring both fascicular and interfascicular matrix were present in the field of view. A set of three matching images were then captured: a grey scale intensity image and two false-coloured images of phase shift (total birefringence) and slow axis orientation. Images were captured using the Rotopol software, and analysed using Gimp 2.8 image processing software (GNU Image Manipulation Program) and ImageJ software (1.50i, National Institute of Health USA).

### 2.3 Tissue birefringence evaluation based on phase shift images

In the context of composites containing birefringent fibres, qPLM allows visualisation of the optical anisotropy resulting from fibre organisation. The first birefringence related imaging channel of qPLM is the phase shift $\left|\sin,\delta \right|$ between the ordinary (fast ray) and extraordinary (slow ray) components of the incident ray after refraction, which is directly proportional to the total birefringence (including contributions from both intrinsic and form birefringence), see Fig. 2c. Assuming a reasonably consistent sample composition (approximately constant intrinsic birefringence), changes in phase shift between samples subsequently indicate a mean degree of out-of-plane structural anisotropy in any particular sample. By contrast, assuming a reasonably constant fibre arrangement, changes in phase shift indicate varying composition of a sample.

Birefringence (total sample birefringence Δn) can be computed from the measured phase shift between the ordinary and extraordinary rays generated as light travels through a birefringent material$\left(\left|\sin,\delta \right|\right)$ and is related to sample thickness (l) and the wavelength of the incident light (λ), as shown in Eq. (1):

(1) $$\Delta n=\frac{\delta \lambda }{2\pi l}$$

By maintaining a consistent value of sample thickness (l, here 5 µm – the histological section thickness) and wavelength of the incident light (λ, here 530 ± 60 nm), the phase shift $\left|\sin,\delta \right|$ can be seen to be proportional to the total birefringence, hence the two terms are often used interchangeably [27]. Therefore for simplicity throughout this paper we referred to the phase shift $\left|\sin,\delta \right|$ images as birefringence images [27], but we computed actual total birefringence values (Δn) from the measured phase shift $\left|\sin,\delta \right|$ using Eq. (1) (see Table 1, Table 2). Moreover, as the qPLM method measures the total birefringence, without quantitative distinction of the contributions from the intrinsic and form birefringence, we referred to the total birefringence as birefringence.

**Table 1:** Total birefringence Δn [−] measured from the phase shift images of unstained 5 µm thick sections of young and old equine superficial digital flexor tendons (SDFTs, n = 6) and common digital extensor tendons (CDETs, n = 6). Measurements were performed in two regions: fascicles and the interfascicular matrix (IFM). Data were normally distributed and show mean ± standard deviation. Age groups were combined as the differences were insignificant with respect to animal age.

Tendon type		SDFT	CDET	Statistical significance
Birefringence Δn [−]	Fascicles	0.0043 ± 0.0005	0.0051 ± 0.0006	*p = 0.06*
	Interfascicular matrix (IFM)	0.0012 ± 0.0001	0.0013 ± 0.0001	*p = 0.2*
	Statistical significance	*p < 0.00001*	*p < 0.00001*	–

**Table 2:** Effects of picrosirius red staining on total birefringence Δn [−]. Matched unstained and picrosirius red stained sections are compared for the equine superficial digital flexor tendon (SDFT, n = 6) and common extensor tendon (CEDT, n = 6). Data were normally distributed and show mean ± standard deviation. Age groups were combined as the differences were insignificant with respect to animal age.

Tendon type		SDFT	CDET	Statistical significance
Birefringence Δn [−]	Fascicles unstained	0.0043 ± 0.0005	0.0051 ± 0.0006	*p = 0.06*
	Fascicles picrosirius red stained	0.0078 ± 0.0009	0.0115 ± 0.0013	*p < 0.00001*
	Statistical significance	*p < 0.00001*	*p < 0.00001*	–
Factor of birefringence increase in matched stained sections		1.8 (±0.4)×	2.3 (±0.8)×	–

The phase shift information was contained within the values of the red channel of the RGB (red-green-blue additive colour model) images obtained from the Rotopol software, and subsequently transformed to grey scale with values ranging from 0 to 255 [3]. Artefacts were removed by an automatic threshold in the Rotopol software, to avoid considering edges of a sample containing no tissue. This threshold removed 0.5% of the minimum and maximum birefringence values and set them to 0 (black). Images were then thresholded in order to separate birefringence in fascicles and the IFM. The threshold levels used for distinguishing birefringence of fascicles and IFM were set to 1–29 for the IFM and 30–255 for the fascicles. Threshold values were means defined and based on the grey scale (intensity) images, ensuring a clear distinction between fascicles and the IFM that is not related to the birefringence or slow axis orientation depicted in the two other modalities of the method. Quantification of the interfascicular matrix birefringence was performed in the unstained sections images only, as in some stained sections, birefringence in the IFM regions was removed by the automatic threshold. Finally, the grey scale range of 0–255 was transformed to a 0–1 scale of sinusoidal phase shift $\left|\sin,\delta \right|$ and birefringence (Δn) was calculated using Eq. (1).

### 2.4 Crimp angle and length evaluation based on slow axis images

The second birefringence related imaging channel of qPLM is the slow axis angle measurement, which denotes the refraction angle of the slow ray (or extraordinary ray direction) through the material, and reflects the in-plane ordering within a section, see Fig. 2d. It effectively offers a 2D projection of the 3D arrangement of fibres in a section.

The total birefringence is related to the orientation angle of the slow axis of a material (φ) in the following manner:

(2) $$\Delta n\approx \left(n_{e},-,n_{o}\right){\left(\sin\varphi \right)}^{2}$$

where n_e_ and n_o_ are the refractive indices in the direction of the extraordinary (slow) ray and the ordinary (fast) ray.

**Fig. 3:**
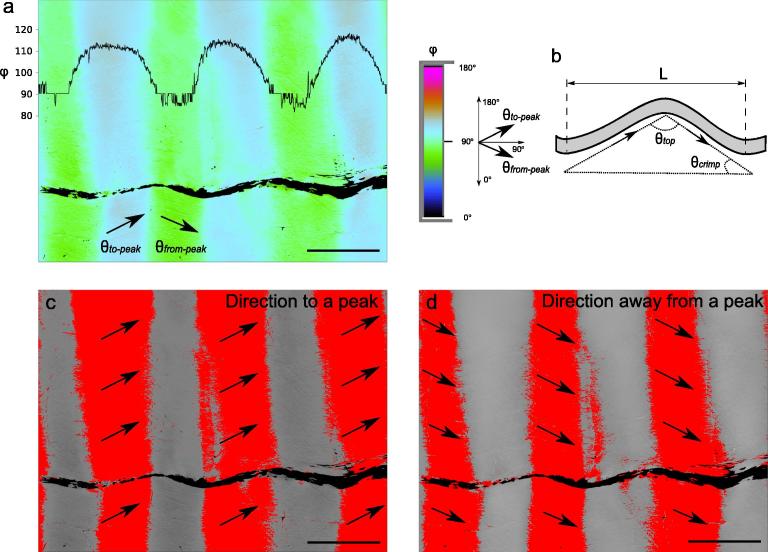
Crimp characteristics evaluated in slow axis images: a) an unstained section of a young CDET, with a black line representing a line plot of slow axis angles superimposed on the image, b) definition of collagen crimp length L and crimp angle θ_crimp_, c–d) thresholding procedure selecting slopes of collagen crimp travelling to and away from a crimp peak. Scale bars = 100 µm.

Slow axis images were utilised for quantitative evaluation of collagen crimp angle θ_crimp_ and crimp length L [13]. Details of the procedures for measuring these parameters are illustrated in Fig. 3.

In order to accurately calculate crimp parameters, it was first necessary to restrict analysis to those samples for which the crimp was in plane with the cutting direction, so no information was lost in out of plane crimp orientation, and the maximal crimp angle was observed in plane. Sections were rarely cut exactly with crimp propagation plane, so most sections showed a small amount of crimp in both the out-of-plane and in-plane directions (birefringence and slow axis images). However, images with clearly out-of-plane crimp that would cause major underestimation of crimp angle, were excluded from the crimp angle analysis. To achieve consistency in the selection of in-plane images, grey-scale thresholding was adopted, whereby out-of-plane images were excluded if crimp pattern (hills and valleys) were still visible in the fascicular region after thresholding to grey scale level of 54 (see Fig. S1 for details of this procedure).

This exclusion resulted in 51% of all sections (61% of SDFT sections and 42% of CDET sections), which showed approximately in-plane crimp, and were used for investigating crimp angle.

Remaining slow axis images were directly transformed from the grey scale range of 0–255 to 0–180° as the potential range of the slow axis orientation. The Rotopol system was calibrated to align the 0˚ direction perpendicular to the long axis of an image (see Fig. 3a). This ensures all pixels in the slow axis images fall within 0–180˚ range. The selected slow axis images were thresholded, initially to ensure they only contain pixels denoting angles in the “to a peak” slope (θ_to-peak_, Fig. 3c) and again to only contain pixels denoting angles in the “from a peak” slope (θ_from-peak_, Fig. 3d). From the averaged value of all pixels selected in the two thresholding operations the two different in-plane orientations of the collagen, as it travelled to and away from a peak (Fig. 3), were determined. The crimp angle (θ_crimp_) was calculated from the two mean in-plane orientations based on simple trigonometry:

$$\theta_{\mathrm{crimp}}=\frac{1}{2}\left(\theta_{\mathrm{to}-\mathrm{peak}},-,\theta_{\mathrm{from}-\mathrm{peak}}\right).$$

The resulting crimp angle is a relative angular measure, which ensures that it is independent of the orientation of crimp wave within the plane of a section.

To assess the extent of error associated with simply estimating crimp angle in regular histological sections, we compared qPLM crimp angle measures with those acquired from a classic manual crimp angle measurement [12]. Here, we took all 39 unstained tendon sections, and measured the angle of 10 randomly selected crimps in each image, acquiring an estimate of mean crimp angle for each image in addition to a mean crimp angle for each test group.

Average crimp length L, independent of sectioning plane, was measured between two adjacent crimp peaks in at least 10 regions of interest in each slow axis image [12].

### 2.5 Statistics

A Lilliefors (Kolmogorov-Smirnov) normality test and quantile-quantile (qq) plots were performed on all data sets, in order to check if data were normally distributed. For data sets that were normally distributed, arithmetic mean values were calculated for the birefringence of thresholded IFM and fascicular regions in each image. Similarly, arithmetic mean of slow axis angles (thresholded: to a peak/away from a peak) were calculated in each image. Mean crimp length was calculated in each image as an arithmetic mean of the length between 10 crimp peaks. Further, means for tendon type (SDFT, CDET), age group (young, old) and staining condition (stained, unstained) were calculated and compared using unpaired two-tailed t-tests for samples with equal or unequal variances. Comparisons of data sets for which normality could not be ensured were performed using the Kruskal-Wallis non-parametric tests. Significance level of α < 0.05 was used.

## 3 Results

### 3.1 Tendon birefringence evaluation based on phase shift images

Total birefringence in tendon tissue is related to the presence of birefringent collagen fibres (intrinsic birefringence), as well as to the extent of alignment of these fibres (form birefringence). When viewing longitudinal tendon sections, the presence of collagen will ensure some birefringence is always present, but increased levels of birefringence denote increased dispersion in the out-of-plane orientation of collagen within the sample. Total birefringence was quantitatively compared between fascicular and interfascicular regions of energy storing SDFTs and positional CDETs of various ages.

#### 3.1.1 Birefringence in unstained tendon sections

**Fig. 4:**
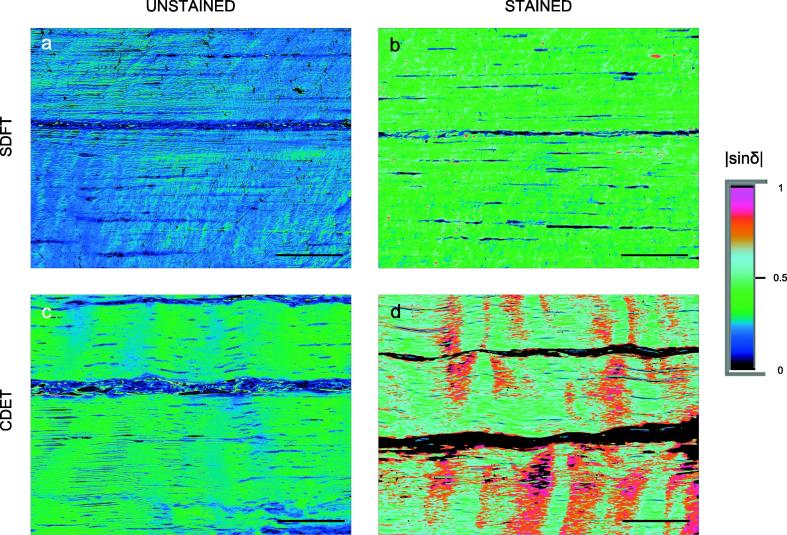
Comparison of matched unstained (a, c) and picrosirius red stained (b, d) sections of a superficial digital flexor tendon, SDFT (a, b) and a common extensor tendon, CDET (c, d). The false-coloured images encode the phase shift |sin *δ*| which is proportional to birefringence (see Eq. (1). Higher birefringence is observed in stained sections (b and d), as compared to the unstained sections (a and c). Scale bars = 100 µm. (For interpretation of the references to colour in this figure legend, the reader is referred to the web version of this article.)

Tendon sections of 5 µm thickness showed sufficient birefringence to be directly investigated under circularly polarised light without staining. Birefringence levels were 0.0040 ± 0.0005 and 0.0042 ± 0.0005 in fascicles of young and old SDFTs, and 0.0058 ± 0.0007 and 0.0042 ± 0.0005 in young and old CDET fascicles, respectively. No significant differences between the age groups were noted in birefringence values for fascicles of either tendon type (p > 0.05), so samples of all ages were combined for further birefringence analysis. The differences between birefringence in fascicles of CDETs (Fig. 4c) and SDFTs (Fig. 4a) were also statistically insignificant, see Table 1.

Average birefringence levels in the IFM of young and old SDFTs were 0.0013 ± 0.0001 and 0.0012 ± 0.0001, respectively and 0.0013 ± 0.0001 in the IFM of both young and old CDETs, highlighting no significant differences with age. Similarly, there were no significant differences in birefringence in the IFM regions of different tendon types, indicating that variations in the dispersion of fibre directions were not tendon specific in either the fascicles or IFM.

In contrast, a comparison of total birefringence levels between fascicles and IFM showed highly statistically significant differences between regions in both tendon types, with significantly lower total birefringence in the IFM than fascicles (Table 1). As qPLM measures the total birefringence, a quantitative distinction of the contributions from the intrinsic and form birefringence cannot be made. Nevertheless, the large differences between the mean birefringence in fascicles and the IFM support the finding that both compositional and structural differences are evident between the two phases of tendon morphology.

#### 3.1.2 Picrosirius red staining effects

The comparison of birefringence in picrosirius red stained and unstained sections was performed exclusively in the fascicular regions of tendons. The variability in IFM area fraction between images meant average birefringence levels across a complete section were heavily affected by the presence of the IFM, and in some stained sections, birefringence in the IFM region was removed by the automatic threshold in the image capturing software (as explained in methods section). Choosing fascicular regions only for a comparison of staining effects ensured appropriate analysis of the picrosirius red stain.

As reported in unstained sections, differences in birefringence with age in stained samples were insignificant, so samples were combined for further analysis, and average birefringence data is provided in Table 2.

Higher birefringence was measured in fascicular regions of picrosirius red stained sections relative to matched unstained sections in both tendon types, and these differences were highly significant (*p < 0.00001* for both SDFTs and CDETs). Whilst an initial visual comparison of the matched stained and unstained sections seemed to suggest that birefringence increased homogenously across stained sections (Fig. 4), a quantitative comparison of birefringence levels highlights that the changes occurring with staining were not always uniform. Significantly more birefringence was evident in the fascicles of stained CDET sections compared with stained SDFT sections (*p < 0.00001,* see Table 2), whilst no significant differences were evident in a matching comparison between these tendons in unstained samples (*p = 0.06*). Directly comparing the mean increase in birefringence with staining highlighted an increase of a factor of 2.3 (± 0.8) × for CDETs as compared to 1.8 (± 0.4) × for SDFTs, which may indicate that some components of fascicular matrix in CDETs attract the picrosirius red stain more intensively.

### 3.2 Crimp angle and length evaluation using slow axis images

Images showing the orientation of the slow axis were used to measure collagen fibre crimp angle θ_crimp_ and crimp length L (see Fig. 3 for definitions). Unstained sections were used for these measurements, as they provided sufficient information, although the stained sections could equally have been used.

**Fig. 5:**
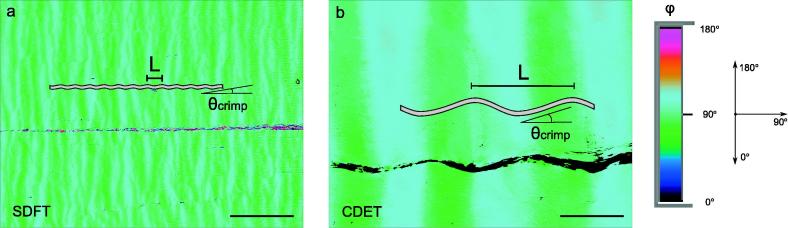
Slow axis orientation images showing crimp length (L) in superficial digital flexor tendon, SDFT (a) and common digital extensor tendon, CDET (b) tendons, as visualised with the false coloured orientation angle of the slow axis images. Notice the crimp length: L is much larger in CDET than in SDFT tendons. Scale bars = 100 µm.

The mean crimp angle and crimp length in fascicles from the SDFT and CDET in the unstained sections are shown in Table 3, and representative images are shown in Fig. 5. Differences between the age groups were not significant for either parameter in either tendon type so data were once again pooled between age groups. However, highly significant differences were evident in crimp angle and crimp length between the tendon types, with SDFTs showing much finer crimp than CDETs (*p < 0.00001*, see Table 3). The crimp height of the CDET was on average 15.7 µm, while crimp was very shallow in the SDFT, measuring 1.2 µm.

**Table 3:** Collagen crimp angle θ_crimp_ [°] at the bottom of crimp wave and crimp length L [µm] (see Fig. 3 for definitions), measured from the slow axis orientation images of unstained sections of SDFT (n = 6) and CDET (n = 6) samples. Data were normally distributed and showed as mean ± standard deviation. Age groups are combined as the differences were insignificant with respect to animal age.

Tendon	Collagen crimp angle θ_crimp_ [°]	Collagen crimp length L [µm]
SDFT	6.5 ± 1.4	21.1 ± 5.5
CDET	13.1 ± 1.8	135.4 ± 20.1
Statistical significance	*p < 0.00001*	*p < 0.00001*

Comparing qPLM data with histological crimp angle measurements identified the variability associated with the classic histological method (see Table S1 for details). Unsurprisingly, without the use of qPLM to identify sections with out of plane crimp, a measure of crimp angle across all histological sections led to an underestimation of mean crimp angles, with mean underestimations of 10% in the SDFT and 45% in the CDET (see Table S1 for details).

In the majority of sections (72%), crimp pattern (length, angle and location of the crimp peaks) was conserved across IFM boundaries, suggesting an order to fascicle structure across multiple fascicles (see Fig. S2a, c). However, in some sections conservation of crimp arrangement was not observed, and neighbouring fascicles had different collagen crimp morphologies and/or varying crimp angles (See Fig. S2b, d). The changing pattern between adjacent fascicles was most commonly seen in old CDETs where crimp pattern was preserved in only 43% of sections, while in the sections from young CDETs, the patterns were preserved in 90% of sections. In SDFTs, crimp patterns were preserved in 63% of sections from young tendons and 86% of sections from old tendons.

### 3.3 Injured tendon birefringence and crimp characteristics

Six unstained sections from a visibly injured SDFT tendon were additionally investigated for birefringence and collagen crimp characteristics.

Mean birefringence measured in the fascicles of the unstained sections of the injured SDFT was 0.0037 ± 0.0003 [−], significantly lower (*p = 0.0003*) than the birefringence levels observed in SDFTs without visible damage (0.0043 ± 0.0005 [−]). This reduction is likely to be partially related to the significantly flatter crimp in the injured SDFT (4.2 ± 0.6°) than the healthy SDFTs (6.5 ± 1.4°, *p = 0.001*). However, it also indicates either more uniaxially arranged collagen fibres, more sparse packing of fibres and/or a change in composition of the injured tendon. The crimp length in the injured tendon (17.6 ± 7.6 µm) was somewhat shorter than that in non-injured SDFT tendons (21.1 ± 5.5 µm), but no significant differences were seen (*p > 0.05*).

**Fig. 6:**
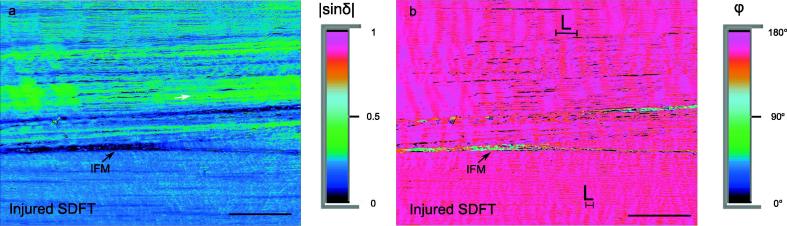
Unstained sections of injured superficial digital flexor tendon, SDFT: a) the false coloured image depicts the inhomogeneity of birefringence (white arrow) within a fascicle from an injured tendon, b) the slow axis orientation image depicts the difference in crimp length L on two sides of the interfascicular matrix (IFM). Scale bars = 100 µm.

Interestingly, variations in crimp pattern and crimp length between neighbouring fascicles were most frequently seen in the injured SDFT sections (Fig. 6), present in 62% of the sections from the injured tendon, compared to 14% of the sections from the healthy SDFTs.

## 4 Discussion

This study quantifies birefringence in fascicles and interfascicular matrix of functionally distinct energy storing and positional tendons. It additionally adopts a novel method to measure collagen crimp angle, avoiding the artefacts related to histological sectioning, and provides direct comparison of the crimp characteristics of functionally distinct tendons of various ages.

A small number of previous studies have reported birefringence of (soft) collagenous tissues in a quantitative manner, resulting in values ranging from approximately 0.002 [−] in 10 µm unstained sections of mammalian cochlear tissue [27] to 0.0034 ± 0.0003 [−] in single collagen fibres from (unstained) mouse tail tendons [44]. However, to the best of our knowledge no quantitative birefringence assessment of histological tendon sections has previously been reported. The birefringence values measured across whole unstained sections of SDFTs (comprising both fascicular and interfascicular matrix) were 0.0045 ± 0.0005 [−], slightly higher than those reported for single collagen fibres of mouse tail tendon, which may indicate the influence of multiple collagen fibres arrangement in tendon sections, resulting in increased values of form birefringence.

Differences in birefringence within fascicles between tendon types were weak, and no significant differences with ageing were evident in either tendon type. However, it was notable that differences in fascicular birefringence between SDFT and CDET fascicles approached significance, and with increased sample numbers, significant differences between fibre arrangements in the two tendon types may become apparent. Nevertheless, the data collected suggests that there is little specialisation in collagen fibre packing or spatial dispersion within the fascicles of functionally distinct tendons and no notable alterations with ageing. As such, specialisation and age changes may be more pronounced in terms of the composition of non-collagenous (and non-birefringent) constituents of tendon (like proteoglycans or elastin) and/or structural changes at other hierarchical levels of the tissue, like the fascicle bundles and/or collagen fibres. For example, it has been suggested that there may be considerable changes in the helical organisation of tendon fascicles, which may not be evident in histological sections [12], whilst changes occurring at the collagen fibril scale will have been averaged and potentially lost with the qPLM technique, as the resolution of the birefringence and slow axis images limits the detection of possible variations in collagen crimp and spatial organisation to approximately 5 µm (section thickness and therefore the z-direction resolution).

By contrast, this study provides the first quantification of clear differences in birefringence levels between fascicular and interfascicular matrix in tendons. Across all samples, mean levels of birefringence were substantially lower in the IFM (0.0013 ± 0.0001 [−]), as compared to fascicular regions (0.0044 ± 0.0005 [−]), confirming considerable variation in birefringence between the two regions. These data match histological and proteomics findings, suggesting a far more sparse fibrillar structure in the IFM and relatively higher amounts of collagen type I in fascicles [2], [6], [7], [45].

Of note, this study also shows no significant differences in IFM birefringence between energy storing and positional tendons, indicating that the arrangement and amounts of birefringent collagen fibres in the IFM do not differ largely between functionally distinct tendons. Recent studies have indicated variations in the amounts of a range of non-birefringent components in the IFM of functionally distinct tendons, such as elastin and proteoglycans (especially lumican [6]). Moreover, other studies have identified changes to elastin with age [46] which would not be detected with qPLM. Taken together with the current data, this provides further evidence for the hypothesis that variations in tendon mechanical properties may arise from differences in non-collagenous matrix components specifically, despite the fact that these components constitute a small percentage of the total tendon structure [8], [9], [10], [11], [47], [48].

It should be noted that elastin, due to its fibrillar arrangement, may contribute to form birefringence [49]. However, it has been shown to have a very minor contribution to tissue retardance, meaning it does not have considerable intrinsic birefringence [22], [50]. As such, the larger amounts of elastin and various proteoglycans which have been identified in the IFM of energy storing tendons, likely facilitating efficient tendon recoil [9], [45], [47], will not be considered by the qPLM technique used in this study. The qPLM method also does not account for the extent of post-translational modifications of collagens [51]. Both the levels of enzymatic cross-links and the advanced glycation end products (AGEs) likely differ in various functional tendon types, between fascicles and the IFM, and/or change with aging [52].

Birefringence in injured SDFT sections was lower than in all other tendons, which could indicate one of three possible variations: more sparse packing of collagen fibres within fascicles, lower volume fraction of birefringent fibres in the light path, or more aligned collagen fibres, showing lower dispersion of angular orientation. Other histological studies, along with the evidence of increased inhomogeneity in birefringence within fascicles would all suggest increased collagen fibre alignment is highly unlikely in injured tendons. However, lower collagen fibres volume fractions could cause a loss of mechanical properties and abnormal loading of tendon fascicles, that may in turn lead to damage, degradation and/or injury [7].

This study provides the first systematic data on collagen crimp parameters in functionally distinct equine tendons of various ages. Caution must be taken when comparing the crimp length data across the literature, as there exist a variety of crimp length definitions. Some authors define the crimp length as the length between a crimp wave’s peak and base (“true crimp length”) [53], while still measuring the crimp length as a half of L, as defined in Fig. S3, ie. the half-distance between the adjacent crimp peaks. This type of definition requires a computation of the “true crimp length” using the average crimp angle, and may contribute to the variability in crimp length values cited in the literature and the differences in the age-related trends observed.

**Fig. 7:**
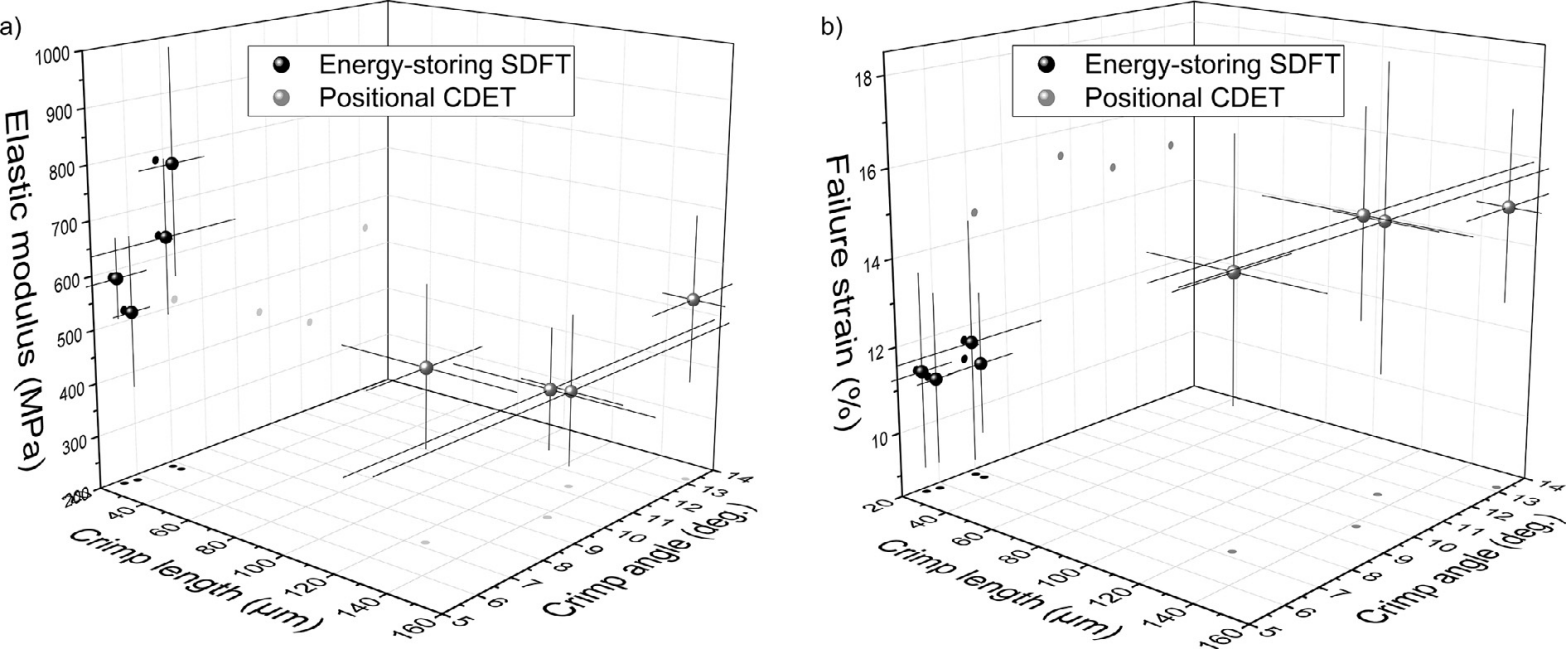
Crimp parameters (length and angle) related to the mechanical properties of fascicles: a) elastic modulus and b) failure strain. The data represents mean values (with bars representing standard deviations) of eight tendons (of 12 tested with qPLM) for which a matching experimental data has been acquired in a study by Thorpe et al. [9]. Dots represent 2D projections of points into the x-y and y-z planes.

In agreement with previous studies [12], [53], [54], [55], [56], we found that crimp was much finer in SDFTs (21.1 ± 5.5 µm) than CDETs (135.4 ± 20.1 µm), a factor that may influence the mechanical properties of the fascicles and be profitable in terms of increasing early extensibility and recoil in energy storing SDFT tendons. Indeed, in a previous study, uniaxial tensile tests have been carried out in isolated fascicles from 8 of the 12 tendons examined in this study [9]. Reviewing all data from this previous study indicates that the elastic modulus of energy storing SDFT fascicles was higher (638 ± 172 MPa) than seen in CDET fascicles (461 ± 157 MPa), with significant differences between tendon types (*p < 0.05*), but no significant differences between age groups. Further, direct comparisons of crimp parameters and mechanical properties can be carried out in the eight matched samples, demonstrating clear trends of higher stiffness in SDFTs (638 ± 172 MPa) associated with smaller crimp angles (6.5 ± 1.4˚) and crimp lengths (21.1 ± 5.5 µm), as compared to CDFTs with a lower stiffness (461 ± 157 MPa) associated with a larger crimp angle (13.1 ± 1.8˚) and crimp length (135.4 ± 20.1 µm), see Fig. 7a. Further samples are required to carry out more detailed correlative analyses.

CDET fascicles, that showed a larger crimp angle and crimp length, were seen to fail at higher strains (15.0 ± 2.1%) than SDFT fascicles (11.3 ± 2.6%) [9] on average (Fig. 7b). Together, data indicated that the larger crimp in CDET fascicles may lead to a greater “hidden” length to these fascicles, reflected in the higher failure strains in this tendon type [9]. However, the differences in crimp length between the tendon types may additionally be attributed to other factors, such as anatomical location or tendon length.

Interestingly, in this study we have found no significant differences in crimp length between skeletally mature young and old horses, which suggests there may be few changes in collagen crimp length after animal skeletal maturation. While this contrasts with previous studies, which have indicated crimp length changes with aging, it is notable that there is little consistence between studies: as both shortening [53] and lengthening [57] of crimp with age have been reported. Crimp length measurements in qPLM images are performed in the same manner as during normal histological examination of sections, therefore this difference cannot be attributed to the method itself. However, data may reflect differences in sample preparation, such as different tissue fixation protocols, that may influence the tissue pre-stress state differentially in young and old tendons. Furthermore, it has also been shown that crimp length is affected by loading history, with a lower crimp angle and length observed in exercised as compared to control horses, likely a result of a fast remodelling process and more newly laid collagen in the exercised horses [58]. The loading history is unknown for the tendons used in this study, so ageing differences may also be masked by variation in loading history.

Indeed, it has been hypothesised that crimp length in tendon may not be directly associated with animal age, but more specifically with the tissue age, with a finer crimp in newly laid fibrils [56]. Indeed, it has previously been shown that crimp is much shorter in injured rat tails, where younger regions of matrix have been newly laid down or remodelled after damaged tissue has been resorbed [56], [57], [59]. The current data does support this; in the injured SDFT tendon (six sections from a single tendon), we measured a shorter crimp length of 17.6 ± 7.6 µm versus 21.1 ± 5.5 µm measured in healthy SDFTs. Whilst only in a single tendon, so not sufficient to draw substantive conclusions, the shortened crimp in the damaged tendon may be attributed to newly synthesised collagen, or may be related to a loss of collagen natural pre-stress in response to regions of damage and stress deprivation, leading to crimp tightening and impaired mechanical performance [60], [61], [62]. Indeed, local damage and a loss of pre-stress has been hypothesised to lead to tendinopathy through the under-stimulation of resident tendon cells [63].

Whilst crimp patterns were often preserved across the IFM boundary between fascicles, some samples in all tendon groups demonstrated altered crimp patterns across two adjacent fascicles. It is possible that these inhomogeneities occur naturally, but they may also be a result of damage and repair within the tendon. Indeed, they were observed more frequently in the injured tendon samples but also in young SDFT and old CDET samples. The more frequent discontinuities across fascicle boundaries seen in young relative to old SDFT samples may be indicative of a higher remodelling rate in the younger group, whilst more discontinuities in old than young CDET samples may be indicative of more damage accumulation with age in CDETs. Further investigation of these changes would be of interest for future studies. Inhomogeneous regions of crimp may pose certain mechanical consequences, creating inhomogeneous damping across the tendon, and increasing the degree of shear within the IFM during normal tendon use. As such, it may be hypothesised that inhomogeneous crimp patterns increase the risk of further tendon damage with use. Further, no gradual transition zone between fascicles and interfascicular matrix was observed, but rather a sharp transition between the zones was seen. This certainly has consequences for the mechanical behaviour of tendons and is important for prescribing proper boundary conditions for modelling tendons mechanical behaviour [64], [65], [66].

While measures of collagen crimp length in 2D longitudinal histological sections are independent of the cutting plane, the crimp angles seen in these sections are strongly dependent on the relative orientation of the sectioning plane to the crimp propagation plane, and subsequently subjected to underestimation errors [22]. The qPLM technique used in this study overcomes this limitation, allowing the relative alignment of the sectioning plane and crimp propagation plane to be assessed in the birefringence (phase shift) images, so crimp angle can be measured only in images cut approximately along the crimp plane.

Crimp angle was lower in the energy storing SDFT (6.5 ± 1.4°), as compared to the positional CDET (13.1 ± 1.8°) and this dependence was not significantly affected by age. This finding was surprising, and does not support previous results showing crimp flattening with age [57], [58]. However, with previous studies identifying exercise history as a primary factor influencing the reduction in crimp angle with ageing [58], the putative lack of exercise history in the current study may contribute to the lack of a significant difference.

In the literature, crimp angles measured from traditional histology images of equine SDFT tendons have ranged from 4.6 ± 0.1° to 14.3 ± 0.4° [12], [58], [67]. To better facilitate a comparison between the current images and previous data, crimp angles in these samples were additionally measured using the classically adopted approach. As anticipated, lower average values for crimp angles were obtained when the cutting plane artefact was not taken into consideration (see Table S1), meaning crimp angles in the current samples are at the small end of the reported range. The low crimp angle in SDFTs may be necessary in order to obtain a greater fibre packing density within a fascicle. Moreover, the fascicles of energy storing tendons have previously been shown to be arranged in a helical fashion, and the densely packed fascicles with flatter crimp in the SDFT may offer a packing arrangement more suited to rotation.

Little previous data depicting the crimp characteristics of equine CDETs are available, and that available is limited to young, skeletally immature horses (1.5 years old). However, the average crimp angle of these young CDETs was 7.0 ± 1.0° [12], which matches well with the manual histological measurement data collated in this study (7.3 ± 3.2°). However, after exclusion of sections with clearly out-of-plane crimped fibres, the mean corrected qPLM crimp angle measurement in the same sections result in 13.1 ± 1.8°. It was notable that the correction resulted in the exclusion of a larger number of sections from CDETs and also resulted in a far larger adjustment to the mean crimp angle. This may be associated with the crimp height exceeding the thickness of histological sections in this tendon type specifically and/or related to the differences in fibre diameters in SDFTs and CDETs [68]. These data further highlight the need for caution in defining crimp parameters from standard histological sections, as the effects of sectioning plane may not influence all tendons evenly.

Indeed, the observed combination of collagen crimp angle and length data from the energy storing and positional tendons highlight how much greater crimp height is in the CDET. This is surprising, as a previous study of functionally different rat tendons (the positional rectus femoris tendon and the energy storing vastus intermedius tendon) reported a similar crimp height for the two tendon types (13.1 and 9.9 µm, respectively) [69]. The crimp height of the positional CDET in the current study was slightly greater than these values (on average 15.7 µm), but comparatively, crimp was very shallow, in the energy storing SDFT (1.2 µm). In a previous study of young (1.5 years old) horses, crimp height differed between tendon types by only a factor of approximately 1.6× [12], with values of 3.4 µm and 5.5 µm for the SDFT and CDET, respectively. The current data suggest that tendon crimp characteristics (crimp length, angle and the resulting crimp height) further specialise to specific tendon function with maturation. Shallow crimp may enable easier packing of collagen fibres within a fascicle and therefore facilitate the energy storing function of tendons.

As the qPLM technique enables crimp orientation to be assessed relative to section cutting plane, it also provides additional insight into the question of crimp organisation as either a planar or helical structure. Within the field of view of current images (at 20× magnification), no clear change in crimp orientation relative to sectioning plane is evident, suggesting that either crimp is a planar wave [38], or that the angle of any fibril level helix is small. Certainly the current data do not clearly show a 3D helical wave to collagen crimp, as has previously been hypothesised [40].

Previous studies have used picrosirius stained soft tissue sections to investigate composition and arrangement of fibres in collagenous structures [25], [26], [29], [30], [31], [41], nevertheless this is the first to document quantitative effects of this stain on tissue birefringence. As expected, we found a clear increase in fascicle birefringence in stained sections as compared to matched unstained sections, with an average fold change of 2.1 (± 0.7)×. However, whilst this correction factor, independent of section thickness, could be used to approximate birefringence of native tissue from stained sections, caution must be advised, as data indicate that information is accentuated with staining in an inhomogeneous manner. A comparison of birefringence in the fascicular region of matched stained and unstained tendons sections, identified non-homogenous staining between tendon types with picrosirius red, and leads us to recommend that only unstained sections are analysed in this manner. It is not possible to tell if the inhomogeneity is a result of the staining, or the qPLM technique adopted. As the measured phase shift is a sinusoidal function ($\left|\sin,\delta \right|)$ establishing an absolute value (the fringe) is not straightforward, and qPLM usage should be restricted to measuring relatively low levels of phase shift (birefringence). The low birefringence levels in unstained 5 μm-thick samples provide confidence birefringence data can be used directly. However, in picrosirius red stained tendon sections, birefringence is substantially higher, hence the risk of recording birefringence values from higher fringes of the sinusoidal phase-shift-function cannot be directly excluded. Further studies incorporating qPLM in stained tissues could take advantage of multi-wavelength measurements [3], [70] to distinguish the fringe of the phase shift measured.

This study demonstrates the use of quantitative polarised light microscopy (qPLM) to assess birefringence and the arrangement of collagen fibrillar structures in soft tissues. We report quantitative information on the structural differences between the functionally distinct tendons. Namely we demonstrate significant crimp length and crimp angle differences between the energy storing SDFT and positional CDET. Importantly the qPLM technique enables assessment of collagen crimp avoiding artefacts related to regular histology measurements.
