# Supplementary material for: Structure and collagen crimp patterns of functionally distinct equine tendons, revealed by quantitative polarised light microscopy (qPLM)
Source: Acta Biomater. 2018 Apr 1;70:281–92. doi: 10.1016/j.actbio.2018.01.034 (PMC5894809; doi:10.1016/j.actbio.2018.01.034)
Supplement: Supplementary data 1 [file mmc1.docx]

**Supplementary materials**

*
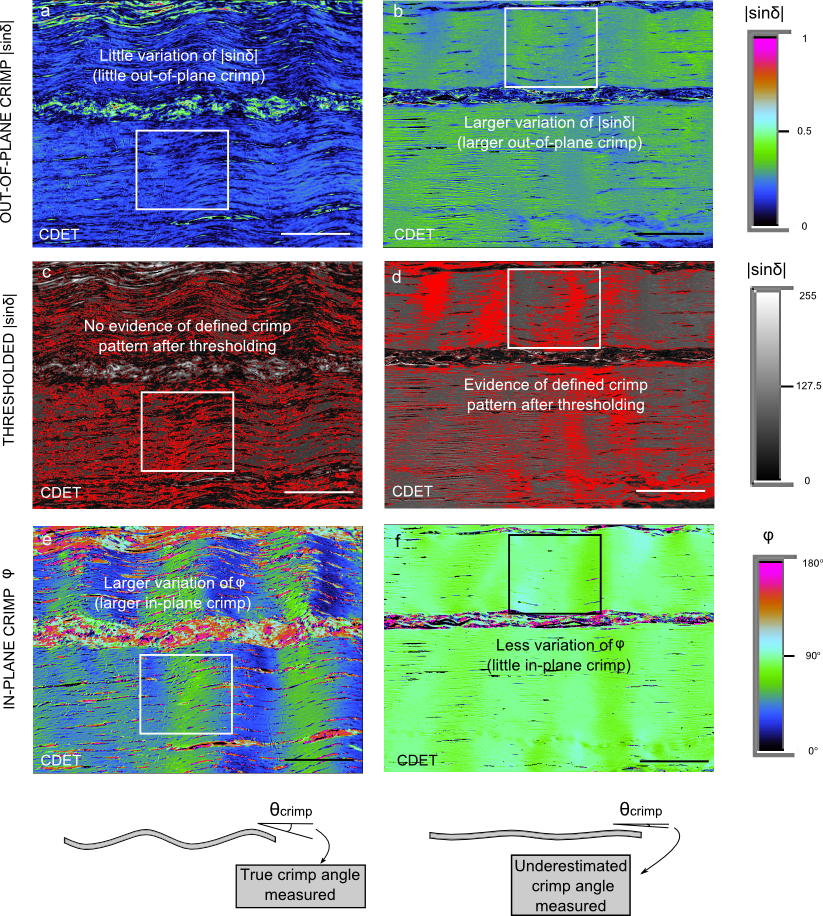
*

*Fig. S1. Representative images showing the selection procedure for evaluation of crimp angle in CDET tendons. The two modalities of qPLM enable observations of crimp propagation both in- and out-of-plane, which allows selection of sections in which crimp propagates approximately in plane. To ensure accurate measure of crimp angle, in-plane crimp should be large, with little or no crimp visible in the out-of-plane direction, as shown in the panels to the left of the figure.*

*In order to quantify the out-of-plane crimp and ensure an objective cut off for rejecting images, grey-scale* $\left| \sin\delta\right|$ *image thresholding was adopted at the cut-off level 54, with images in which crimp was still visible after thresholding discarded from later crimp angle measurements. Rows 1 & 2 show images of the out-of-plane crimp before (a & b) and after (c & d) thresholding, in which it is evident that crimp is only visible after thresholding in d, where out of plane crimp is substantial. Crimp measures would only be taken from e) where crimp is visible, and image f) discarded as only a projection of crimp is visible. Scale bars = 100 µm.*


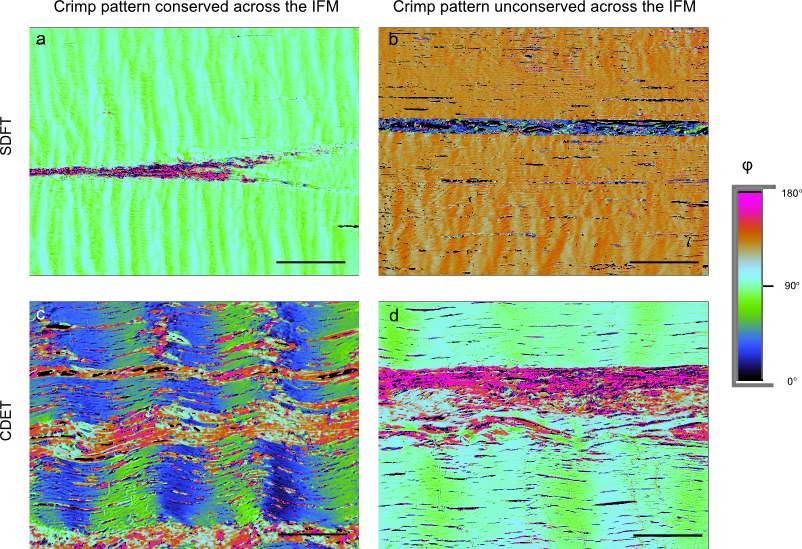


*Fig. S2.* *Representative slow axis images showing crimp patterns across IFM boundaries: a) SDFT section with a crimp pattern clearly preserved across the IFM, b) SDFT section where crimp shows distinct patterns across the IFM boundary, c) CDET section with clearly preserved crimp patterns across multiple IFM boundaries, d) CDET section showing distinct crimp patterns in fascicles separated by IFM. Scale bars = 100 µm.*


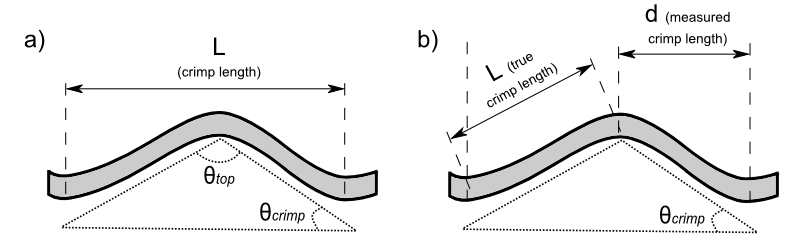


Fig. S3. A comparison between the crimp length definitions present in the literature: a) crimp length defined as a distance between two adjacent crimp peaks (or bottoms), b) crimp length (true crimp length) defined as the length along crimp wave from crimp bottom to crimp peak, as well as the measured crimp length, defined as half-distance between two adjacent crimp peaks (or bottoms). The definition for crimp angle (θ_crimp_) is consistent in the literature.

| Tendon | Collagen crimp angle θ_crimp_ [ᵒ] | | | |
| --- | --- | --- | --- | --- |
|  | qPLM  all sections | qPLM  sections with approximately in-plane crimp | Classic histology  all sections | Classic histology  sections with approximately in-plane crimp |
| SDFT | 5.9 ± 1.2 | 6.5 ± 1.4 | 5.8 ± 1.8 | 6.3 ± 2.4 |
| CDET | 9.3 ± 4.1 | 13.1 ± 1.8 | 7.3 ± 3.2 | 8.8 ± 3.4 |

*Tab. S1. Comparison of collagen crimp angles measured with qPLM and classical histology in all sections as well as in sections with approximately in-plane crimp (selected with qPLM birefringence imaging). The key comparison of classic histology with qPLM is highlighted in green. Collagen crimp angle* θ*_crimp_* *[ᵒ] was measured at the bottom of crimp wave (see Fig. 3 for definitions). Data were normally distributed and shown as mean ± standard deviation (n =6 per tendon type). Age groups are combined as the differences were insignificant with respect to animal age.*
